# TOPOS: target organ prediction on scout views for automated CT scan planning

**DOI:** 10.1007/s00330-026-12623-3

**Published:** 2026-05-14

**Authors:** Sebastian Ziegelmayer, Tristan Lemke, Markus Graf, Su Hwan Kim, Lukas Lemke, Christian J. Mertens, Felix Busch, Dominik Weller, Marcus R. Makowski, Lisa C. Adams, Keno K. Bressem

**Affiliations:** 1 Department of Diagnostic and Interventional Radiology, Klinikum rechts der Isar, Technical University of Munich, School of Medicine and Health, TUM University Hospital, Munich, Germany; 2 Department of Cardiovascular Radiology and Nuclear Medicine, Technical University of Munich, School of Medicine and Health, German Heart Center, TUM University Hospital, Munich, Germany

**Keywords:** Tomography, X-ray computed, Deep learning, Radiography, Radiation dosage, Image processing, computer-assisted

## Abstract

**Background:**

Overscanning is a common issue in CT planning, leading to unnecessary radiation exposure.

**Purpose:**

To develop a deep learning model to segment anatomical structures in scout views to optimize scan ranges and reduce radiation.

**Materials and methods:**

In this single-center retrospective study, 1146 patients undergoing CT between 2022 and 2025 were included. The model was trained on segmentations of 26 target structures in five regions (head, neck, chest, chest-to-pelvis, abdomen-to-pelvis), transferred to scout views and manually corrected. Performance was evaluated using the Dice-Sørensen coefficient and normalized surface distance on an internal test set of 100 patients and 36 external chest CTs. Automated versus manual scan planning was compared in 61 internal (chest, upper abdomen, head) and 14 external (chest) CTs, with z-axis coverage and dose-length product.

**Results:**

1146 patients (mean age, 63 ± 17 years; 577 men) were included. For target structures in five regions, mean DSC and NSD were 0.93 and 0.88. External mean DSC across chest targets was 0.851 ± 0.051. Automated planning captured relevant anatomy in 98% of internal and 92.9% of external CTs. Scan length significantly decreased for automated planning in the internal test cohort (chest 50 mm (15%), *p* < 0.001; upper abdomen 60 mm (25%), *p* < 0.001; cranial 19 mm (11%), *p* < 0.001), yielding corresponding DLP reductions of 19%, 25% and 11%, respectively. In the external cohort, scan length decreased by 115 mm (28.7%, *p* < 0.0001) with a corresponding 28.5% DLP reduction.

**Conclusion:**

The proposed model enables reliable automated CT scan planning and reduces overscanning and radiation exposure without compromising diagnostic quality.

**Key Points:**

***Question*** *Can segmentation of anatomical structures on CT scout views enable automated scan planning to reduce overscanning and unnecessary radiation exposure?*

***Findings*** *A deep learning model segmented planning-relevant anatomy on CT scout views and reduced scan length and dose while preserving anatomical coverage.*

***Clinical relevance*** *Unnecessary radiation from overscanning is a patient safety concern. Automated CT planning with the proposed deep learning model reduces radiation exposure while ensuring full anatomical coverage for the evaluated scan regions.*

**Graphical Abstract:**

**Figure:**
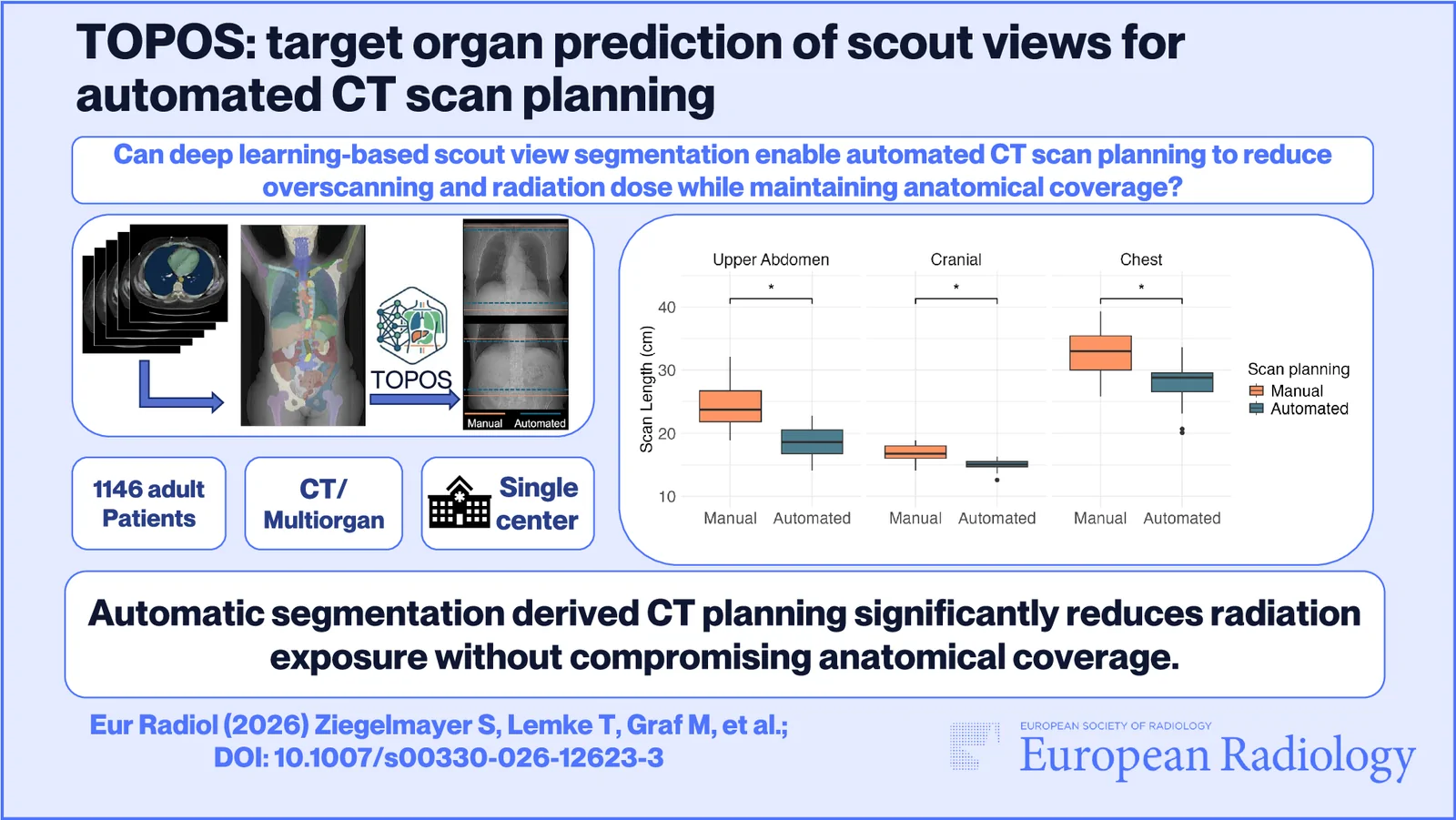

## Introduction

Computed tomography (CT) has become the cornerstone of modern diagnostic imaging, with examination volumes steadily increasing over recent decades [1–3]. This widespread adoption has made CT the predominant source of medical radiation exposure worldwide [4]. Although CT examinations account for only 9% of all examinations using ionizing radiation, they contribute up to 60% of the radiation exposure [5]. Therefore, continuous development and implementation of effective dose-optimization strategies for CT are crucial to reduce unnecessary radiation exposure.

One key parameter affecting radiation exposure is the scan length, defined as the anatomical region to be examined. Radiographers manually determine the scan length by referencing frontal or lateral overview radiographs, commonly referred to as scout view radiographs, but also as topograms, scanograms, overview radiographs or localizer radiographs. We use the term scout view throughout this work, reflecting common usage in the literature. As radiation exposure directly correlates with the scan length, scan-range optimization tailored to the clinical question can substantially reduce the radiation dose [6–8]. However, uncertainty regarding accurate coverage of the region of interest frequently results in precautionary manual extension of the scan range, leading to manual overexpansion of the scan field. As a result, overscanning occurs in 30–60% [9, 10] of cases in clinical practice and leads to a substantial increase in radiation dose [11, 12]. Moreover, the steadily increasing workload [2, 13], combined with high burnout rates in radiology, creates a self-reinforcing cycle, and studies show significantly higher rates of overscanning in high-throughput settings [10] and among less experienced radiographers [9].

Artificial intelligence (AI) offers potential solutions to these challenges and has already been successfully applied to various aspects of CT imaging, including patient positioning, protocol selection, denoising, and image reconstruction [14–17]. However, the automatic delineation of optimal scan ranges using AI has been minimally explored, with existing studies limited predominantly to the chest region [18–20]. To address this gap, the present study aimed to develop and validate an AI model capable of accurately segmenting multiple anatomical structures critical for determining scan ranges in scout views across diverse body regions and subsequently evaluate its efficacy in automated CT scan planning.

## Materials and methods

### Study design

**Fig. 1:**
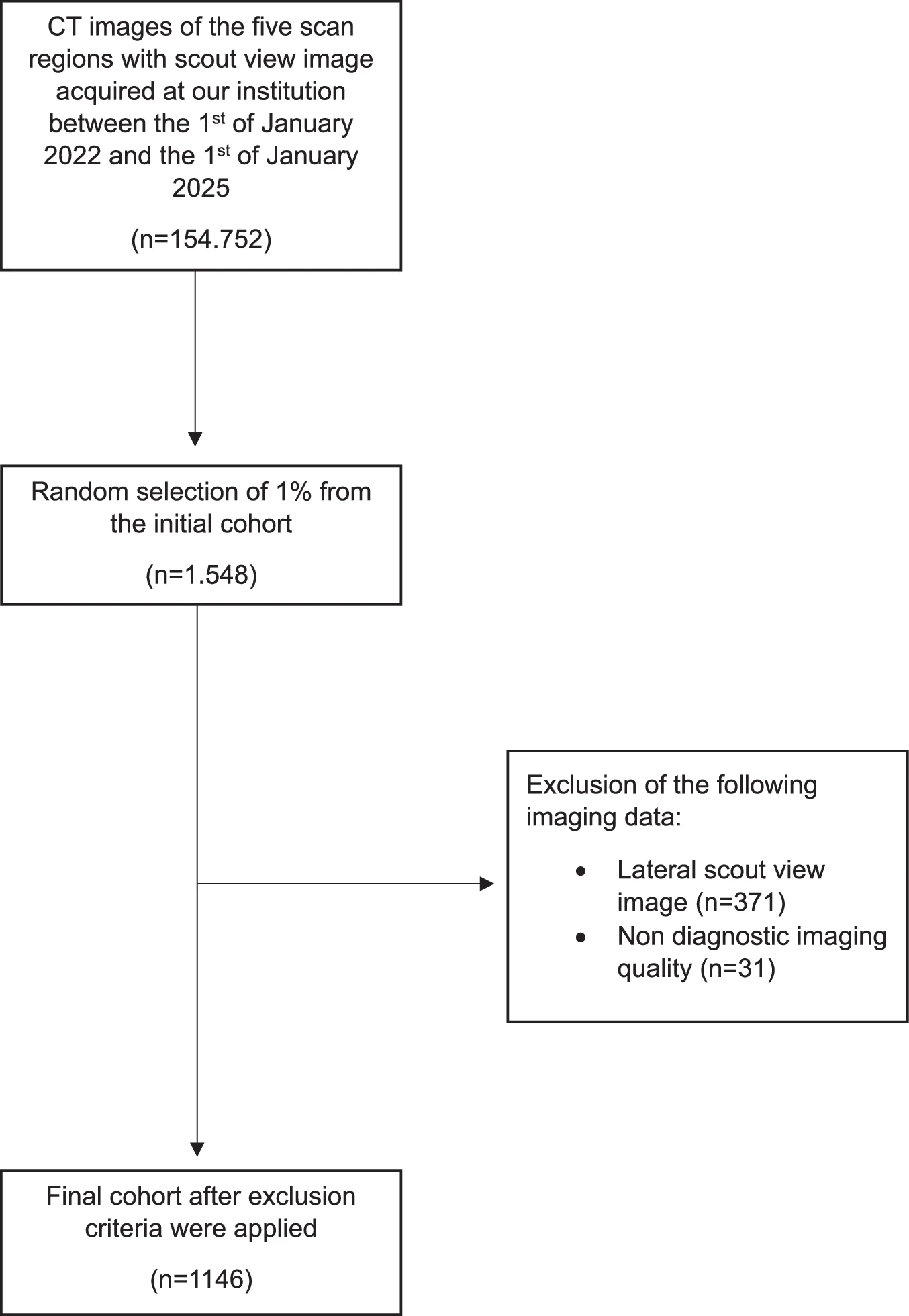
Flowchart illustrating the patient inclusion and exclusion process

This single-center retrospective cohort study analyzed CT examinations performed between January 1, 2022, and January 1, 2025. Approval by the local Institutional Review Board was obtained, and informed consent was waived due to the retrospective study design. The target population comprised all adult patients (age > 18 years) who underwent CT imaging of the head, neck, chest, chest-to-pelvis, and abdomen-to-pelvis regions during the study period (*n* = 154,752). From this eligible population, a random subset of 1548 examinations was selected using a pseudo-random number generator implemented in Python to ensure unbiased case selection. Patients were then excluded if (1) the scout image was in lateral projection or (2) the imaging quality did not meet diagnostic standards, which was determined by a board-certified radiologist (L.C.A., 9 years of experience). After exclusion, the final cohort contained 1146 patients. The patient inclusion process is visualized in Fig. 1, and Patient characteristics are shown in Table 1. To evaluate the model’s segmentation performance across scan regions, we assembled test cohort A from 100 cases selected at random with stratification by anatomical region, resulting in a distribution broadly similar to the training set while intentionally enriching underrepresented regions (head, neck and chest). Test cohort B, consisting of chest and upper abdomen scans with 23 cases each and 15 cranial cases, was used to evaluate the performance of the deterministic scan-range selection procedure based on the model-generated segmentation masks, including z-axis coverage accuracy and radiation dose reduction. The separation into two independent test cohorts was chosen to decouple technical segmentation performance from downstream clinical evaluation and to reduce the risk of selection bias toward anatomically simpler cases when assessing scan-range prediction and dose reduction. There was no patient overlap between the train and test datasets.

**Table 1:** Characteristics of study participants

Characteristic	Training cohort (*n* = 985)	Test cohort A (*n* = 100)	Test cohort B (*n* = 61)
Sex			
Male	519 (54.2%)	58 (58%)	34 (55.7%)
Female	466 (47.3%)	42 (42%)	27 (44.3%)
Age (years)			
Mean ± SD	63.1 ± 16.9	63.6 ± 16.0	64.9 ± 14.2
Scan region			
Head	40 (4%)	10 (10%)	15 (24%)
Neck	125 (12.7%)	10 (10%)	
Chest	131 (13.3%)	25 (25%)	23 (38%)
Chest-to-pelvis	511 (51.9%)	30 (30%)	
Upper abdomen	N/A	N/A	23 (38%)
Abdomen-to-pelvis	178 (18%)	25 (25%)	

*SD* standard deviation

### CT imaging

Imaging data from five different CT scanners were included in this study: Siemens Somatom Definition (64-row, Siemens Healthineers), Siemens Somatom Go Top (128-row, Siemens Healthineers), Philips iCT (256-row, Philips Healthcare), Philips IQon (256-row, Philips Healthcare) and Philips Incisive CT (128-row, Philips Healthcare). The in-plane pixel sizes were: Siemens Somatom Definition 0.977 × 0.977 mm, Siemens Somatom Go Top 0.590 × 0.590 mm, Philips iCT 0.801 × 0.801 mm, Philips IQon 0.977 × 0.977 mm, and Philips Incisive CT 0.742 × 0.742 mm. Table 2 shows the distribution of scans obtained from each CT scanner across the training and both test cohorts. Scout view acquisition parameters across scanners and anatomical regions are summarized in Supplementary Table 2. All scout views that included the chest were acquired during patient inspiration. For each scan, axial CT datasets reconstructed at a 3 mm slice thickness and the corresponding frontal scout view were available in DICOM format.

**Table 2:** Distribution of CT scans from each CT scanner across the training and both test cohorts

Scanner model	Training cohort (*n* = 985)	Test cohort A (*n* = 100)	Test cohort B (*n* = 46)
Philips IQon	576 (58.5%)	68 (68%)	32 (69.5%)
Philips iCT	285 (28.9%)	14 (14%)	7 (15.2%)
Philips IncisiveCT	101 (10.3%)	0 (0%)	0 (0%)
Siemens Somatom Definition	12 (1.2%)	10 (10%)	4 (8.7%)
Siemens Somatom Go Top	11 (1.1%)	8 (8%)	3 (6.5%)

### Image processing and scout view segmentation

The axial CTs and scout views were exported and converted to NIfTI (Neuroimaging Informatics Technology Initiative) format. The generation of the segmentation masks for the scout views was performed sequentially. First, the axial CT images were segmented using TotalSegmentator [21], a robust segmentation algorithm for anatomical structures. The preliminary segmentation masks of 26 structures (Supplementary Table 1) were then reviewed in the scout view and, if necessary, manually refined by a board-certified radiologist. Reasons for adjustment included incomplete coverage of the segmented structure due to undersegmentation in the CT volume or projection errors, oversegmentation with extension into adjacent structures and small misalignments introduced during the projection (e.g., caused by metadata inconsistencies or minor patient motion between scout acquisition and CT scan). For each scan region, target structures were defined that required complete inclusion in their respective CT examinations (Head: Brain, Skull; Neck: Brain, Skull, Cervical Spine; Chest: Aorta, Heart, Lung, Trachea, Thoracic Spine; Abdomen-to-Pelvis: Liver, Kidney right, Kidney left, Aorta, Spleen, Lumbar spine, Colon, Stomach; Chest-to-Pelvis: Aorta, Spleen, Lumbar spine, Colon, Liver, Kidney right, Kidney left, Lung, Thoracic spine, Heart, Stomach, Trachea). Transferring segmentation masks from 3D CT volumes to 2D scout views results inherently in overlapping masks, which must be accurately captured for effective model training. To achieve this, non-background voxels within each segmentation volume were identified, and their 3D coordinates were mapped to 2D coordinates in the scout image space using spatial transformations derived from the scout image metadata. For each anatomical structure, a binary mask was generated on the scout image, representing the projection of the segmentation onto the scout image. Subsequently, morphological operations, specifically including binary dilation and closing, were applied to refine the masks.

### Handling label overlap with prime factorization

**Fig. 2:**
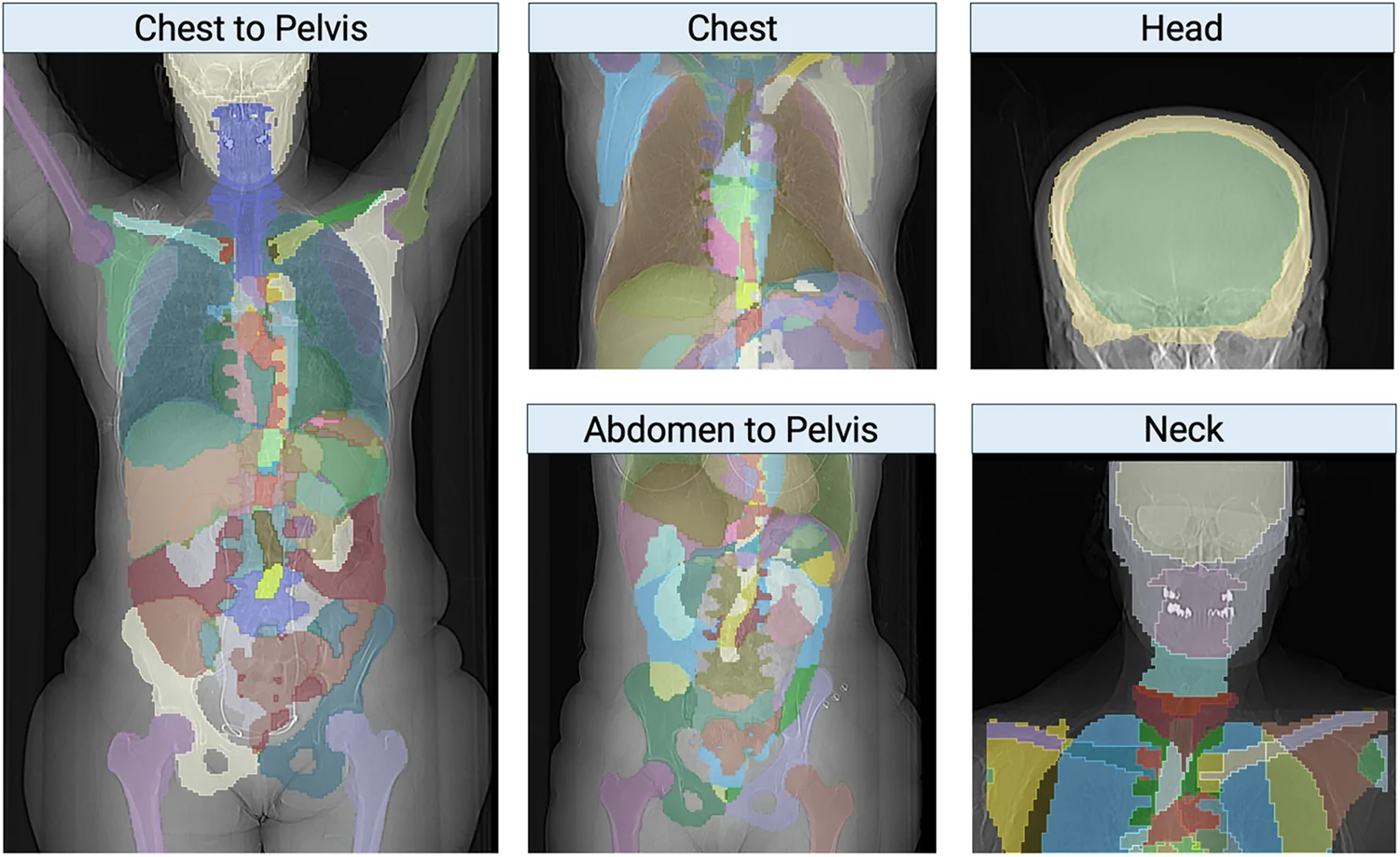
Visualization of AP scout views with segmentation mask for all anatomical labels and their overlap. Each anatomical label is assigned a unique prime number. In regions where multiple structures overlap, a new segmentation mask is computed as the product of the corresponding prime numbers. The resulting composite value allows unambiguous reconstruction of the overlapping anatomical labels through prime factorization

To uniquely identify anatomical structures, each label was assigned a prime number. This assignment ensured that overlaps between multiple structures can be decoded using prime factorization. A bi-directional mapping was established between labels and their primes to facilitate encoding and decoding. To address overlaps where multiple structures occupied the same scout image pixel, the prime number encoding of each structure was multiplied, creating a composite prime value. This composite value unambiguously identified all overlapping labels at each pixel. Global mappings of composite values to their corresponding structures were maintained for model training. Exemplary segmentation masks with the corresponding scout views for the individual regions are shown in Fig. 2.

### Model training and evaluation

For model training, the nnU-Net framework [22] with residual blocks [23] was used. The framework’s automated configuration process determined optimal parameters. To address overlapping labels, a region-based training approach was used, which allows the model to learn area segmentations constructed by merging individual labels. The model was trained for 100 epochs on 985 patients using 5-fold cross-validation, and the best model parameters were retained. Inference was run on the scout views in test cohorts A and B using the optimal model checkpoint, and softmax outputs were saved for the reconstruction of the anatomical label. Segmentation masks of the 26 anatomical labels were generated by assigning pixels exceeding a softmax probability threshold of 0.5 to the respective structure. For test cohort A, the segmentation accuracy was calculated according to standardized recommendations [24], using a 1D Dice-Sørensen Coefficient (DSC) in the craniocaudal axis and the Normalized Surface Distance (NSD) with a maximum tolerated distance of 1 cm. We selected a 1 cm tolerance for NSD to reflect the scale at which deviations in segmentation boundaries could meaningfully influence scan-range planning. In our application, margins of several millimeters are routinely accommodated to account for respiratory motion and anatomic variability; therefore, smaller tolerances are suited to high-precision structure delineation (e.g., radiotherapy planning) but would over-penalize clinically irrelevant edge differences in this context. To assess generalizability beyond our institution, we evaluated our algorithm on the publicly available CPTAC-LUAD dataset, a collection of lung adenocarcinoma cases containing CT, PET, and MR imaging with matched histopathology data (38 subjects, 75 studies) [25]. After manual review, 36 chest CTs from 18 patients were included. Exclusions were due to non-chest CT examinations, fragmented or partially uploaded image series that did not constitute a usable volumetric acquisition, and incomplete anatomic coverage. The included studies comprised contrast-enhanced and non-contrast scans acquired on systems from GE Healthcare (*n* = 20), Siemens Healthineers (*n* = 13), and Philips Healthcare (*n* = 3), including the following scanner models: comprising the following scanner models: GE LightSpeed VCT (*n* = 11), GE Optima CT660 (*n* = 4), Siemens SOMATOM Definition AS (*n* = 4), Siemens Emotion 16 (*n* = 3), GE LightSpeed16 (*n* = 2), Philips IQon Spectral CT (*n* = 2), Siemens Sensation 16 (*n* = 2), and one scan each from GE Optima CT520 Series, GE Discovery 610, GE Revolution CT, Philips Brilliance 16, Siemens SOMATOM Definition AS+, Siemens SOMATOM go.All, Siemens Sensation 64, and Siemens Somaris/5 3D. We applied the identical preprocessing and inference pipeline and computed Dice and NSD for available target structures. The trained model weights and inference code are publicly available at https://github.com/sziegelmayer/TOPOS.

### Scan-range delimitation

The applicability of the model for scan-range planning was analyzed in test cohort B for chest, upper abdomen and cranial scans. The z-axis coverage of the model was defined by segmentation of the target structure for the respective region (CT Chest ~ Lung, CT upper abdomen ~ Liver, cranial CT ~ Skull). To account for scan-range variations due to movement and breathing depth, a 1 cm safety margin was added cranially and caudally (2 cm total). This margin was chosen as a compromise between compensating for patient motion and maintaining precise evaluation of the model’s performance. To compare the scan planning between the model and the manually planned examinations by radiographers, a board-certified radiologist evaluated whether the complete extent of the relevant organs was captured. Insufficient z-axis coverage was marked as erroneous. For adequate scans, both the craniocaudal distance and radiation exposure, measured by the dose-length product (DLP), were calculated.

### Statistical analysis

All statistical analyses were performed using R version 4.2.1 (The R Foundation, http://www.r-project.org). Image and segmentation preprocessing were executed with Python version 3.11. The following patient parameters were recorded for the descriptive statistics: Gender, Age, Region, CTDI, DLP, and number of erroneous scan ranges. DLP for manual and automated scan plans was calculated as CTDI_vol x scan length using CTDI_vol values reported by the scanner; dose reduction was defined as the difference in total DLP between plans. A paired *t*-test was used to compare the scan length and the DLP. A two-sided significance level of *p* < 0.05 was chosen.

## Results

### Cohort characteristics

In total, 1146 patients were included and split into a training set and two test sets. Demographic information and scan region distribution are provided in Table 1. In the training cohort, pleural effusion was present in 19% of chest-containing scans, and atelectasis in 8%. Perihepatic ascites was present in 3% of abdomen-containing scans. No abnormalities relevant to scan-range definition were identified in cranial CT examinations. In test cohort A, pleural effusion was present in 22% of chest-containing scans, and atelectasis in 7%. In abdomen-containing examinations, perihepatic ascites was observed in 2% of cases. Cranial CT examinations in test cohort A did not show abnormalities relevant to scan-range definition. In test cohort B, pleural effusion was found in 25% of chest scans and atelectasis in 10%. In the upper abdominal CTs, right-sided pleural effusion was present in 10% of cases and perihepatic ascites in 15%. For the head region, no abnormalities were found. Typical craniocaudal scan lengths used in routine clinical practice for the training cohort and test cohort A are summarized in Table 3.

**Table 3:** Mean craniocaudal scan length by anatomical region

Region	Training cohort (mean ± SD, mm)	Test cohort A (mean ± SD, mm)
Cranial	148.9 ± 27.9	154.6 ± 30.6
Neck	250.9 ± 33.0	243.0 ± 30.3
Chest	324.4 ± 31.0	333.1 ± 36.2
Abdomen–pelvis	464.5 ± 45.9	471.1 ± 45.1
Chest–abdomen–pelvis	660.6 ± 62.0	656.5 ± 72.1

### Segmentation performance across regions

**Fig. 3:**
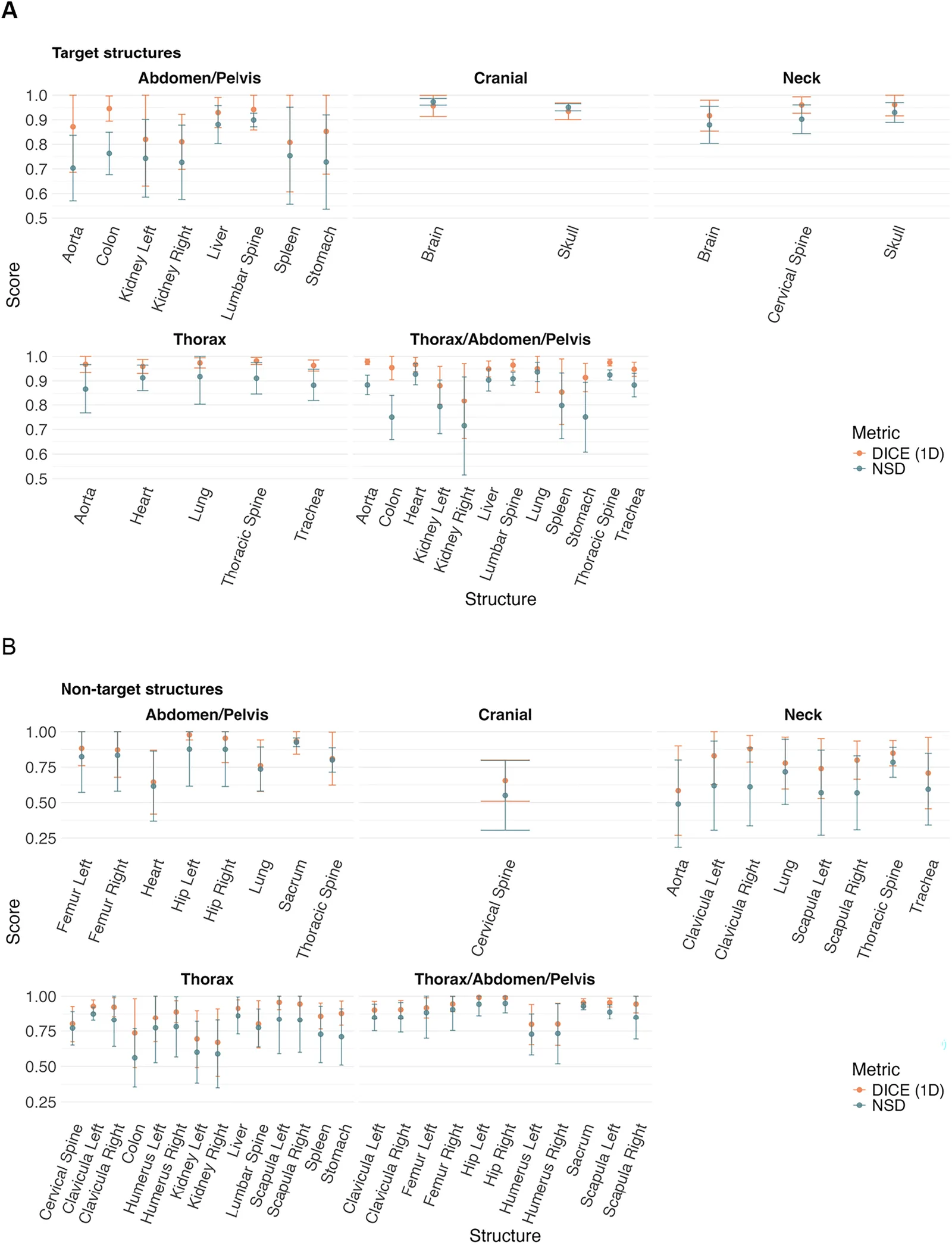
Shown are the DSC (1 D) and NSD of the individual structures for all scan regions. Labels that were not segmented for any of the cases in the individual regions have been removed to improve clarity. The target structures of the individual regions are shown in **A** and the non-target structures in **B**

The model achieved a mean DSC of 0.932 ± 0.060 and NSD of 0.877 ± 0.071 for target structures within the test cohort A. Pooled performance across all regions and structures of test cohort A yielded a DSC of 0.884 ± 0.100 and NSD of 0.801 ± 0.146. On the included studies from the external CPTAC-LUAD dataset (*n* = 36), the model maintained a comparable segmentation performance for available target structures. Mean DSC across the five chest targets (aorta, heart, lung, trachea, thoracic spine) was 0.851 ± 0.051, with a corresponding mean NSD of 0.154 ± 0.038. Region-specific performance for the target structures is summarized in Table 4 and detailed segmentation metrics for individual regions and structures are visualized in Fig. 3.

**Table 4:** Segmentation performance for target and non-target structures, target structures and target structures by region

Region	DSC (mean ± SD)	NSD (mean ± SD) cm
Target and non-target structures	0.884 ± 0.100	0.801 ± 0.146
Target structures		
Overall	0.932 ± 0.060	0.877 ± 0.071
Head	0.946 ± 0.034	0.962 ± 0.014
Neck	0.946 ± 0.047	0.904 ± 0.058
Chest	0.969 ± 0.024	0.898 ± 0.079
Chest-to-pelvis	0.929 ± 0.059	0.848 ± 0.079
Abdomen-to-pelvis	0.872 ± 0.132	0.775 ± 0.128

*DSC* Dice-Sørensen coefficient, *NSD* normalized surface distance

Interestingly, the trained model successfully predicted laterality-dependent structures (heart, spleen, liver) in a scan with situs inversus, suggesting the model acquired true structural features rather than relying on simple spatial localization (Supplementary Fig. 1).

### Automated scan planning of chest, upper abdominal and cranial CT

**Fig. 4:**
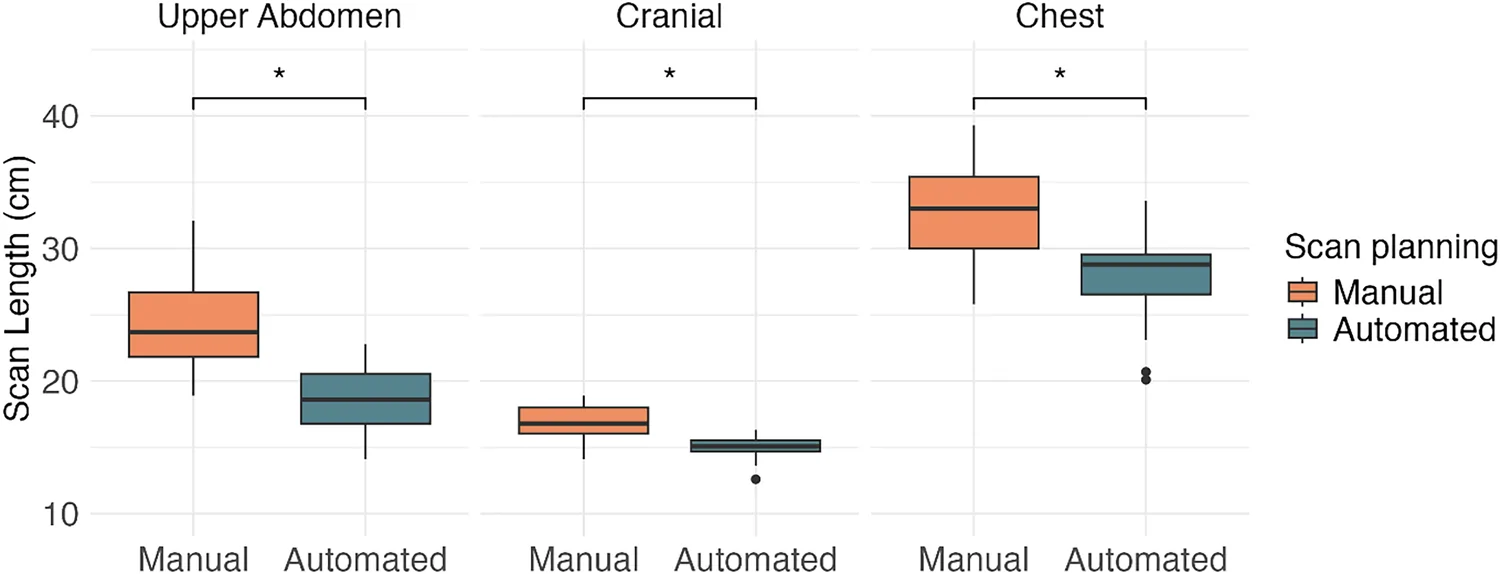
Shown is the scan length for manual and automatic scan planning for chest, upper abdomen and cranial CTs. With automatic scan planning, a significant reduction in scan length can be achieved for all regions

**Fig. 5:**
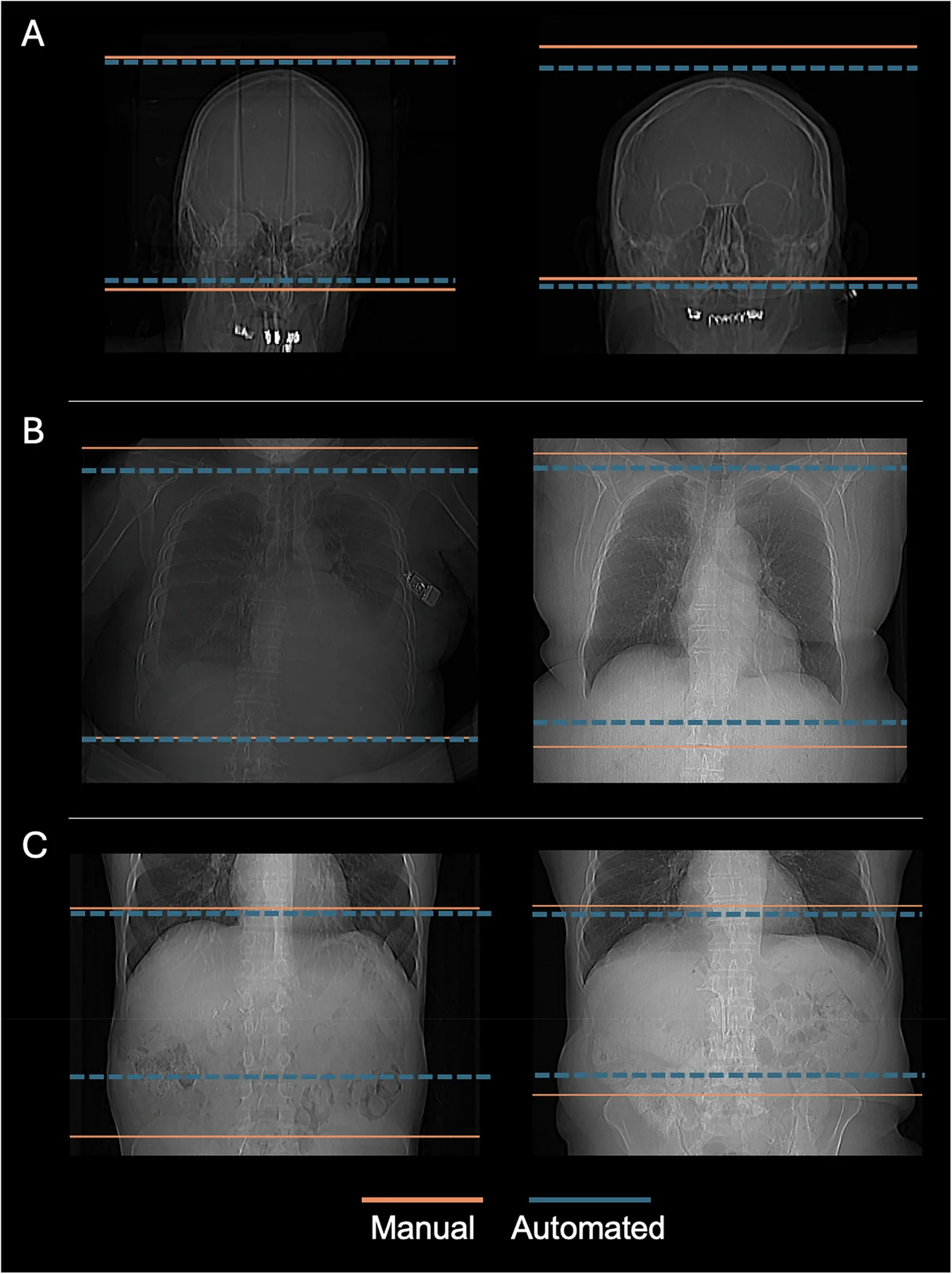
Representative scout views comparing manual and segmentation-derived scan-range planning for cranial (**A**), chest (**B**) and upper abdominal (**C**) CT examinations

The chest scan planning using the lung segmentation did not result in any erroneous CT scans in test cohort B. On average, the automated scan planning led to a reduction in craniocaudal distance of 50 mm (15%) (*p* < 0.001). This enabled a significant reduction in mean DLP from 130 ± 52 to 105 ± 38 mGy*cm, corresponding to a percentage decrease of 19.2%. Upper abdominal scan planning using liver segmentation resulted in 4.35% (1/23) erroneous cases. For the non-erroneous cases, a reduction in craniocaudal distance of 60 mm (25%) (*p* < 0.001) was achieved. This resulted in a significant reduction in mean DLP from 190 ± 63 to 142 ± 45 mGy*cm, corresponding to a percentage decrease of 25.3%. The use of the skull for CT planning of the head resulted in a reduction of scan length by 19 mm (11%) (*p* < 0.001) and a reduction in mean DLP from 623 ± 53 to 552 ± 39 mGy*cm without any erroneous scan planning. In the external evaluation, segmentation-derived chest scan planning was applicable for a subset of the included CPTAC-LUAD studies with available dose information (*n* = 14) and resulted in one erroneous case (7.1%). The craniocaudal scan length for the non-erroneous cases decreased from 40 ± 2.8 cm to 28.5 ± 1.7 cm (mean reduction 115 mm; 28.7%, *p* < 0.0001), with a corresponding reduction in mean DLP from 316 ± 94 to 226 ± 66 mGy*cm (28.5%). Mean CTDIvol values within the internal Dataset were 31.45 ± 2.99 mGy (cranial), 10.30 ± 1.44 mGy (neck), 4.39 ± 2.21 mGy (chest), 8.60 ± 2.52 mGy (upper abdominal), 7.75 ± 1.32 mGy (abdomen-to-pelvis), 6.26 ± 2.57 mGy (chest-to-pelvis). CTDIvol did not differ between manual and automated planning within each protocol. The average scan length for automated and manual scan planning in test cohort B is summarized for different regions in Fig. 4, while representative scout view examples illustrating the corresponding scan ranges are shown in Fig. 5.

## Discussion

In this study, a deep learning model was developed for precise segmentation of multiple anatomical structures in scout views of common CT examination regions. The segmentation of region-specific target structures enables an approach for automated scan planning, leading to a significant reduction in radiation dose compared to manual planning, demonstrated as proof of concept for the chest, upper abdomen and cranial CT.

Since CT is the primary source of artificial radiation exposure, implementing automated scan planning is a promising strategy to reduce radiation exposure. Previous studies, which are exclusively limited to the chest, achieved significant dose reduction while maintaining high accuracy [19, 26, 27] by using anatomical landmarks or lung segmentations. For example, the model by Huo et al [27] achieved a DSC of 0.97 for lung segmentation, though evaluation was limited to a lung cancer screening cohort. Our model was evaluated on a diverse clinical cohort including patients with pleural effusion and atelectasis, which better represents the expected patient population in routine practice. While our model achieved a slightly lower DSC, this did not impact scan planning accuracy. Even in challenging cases, no planning errors occurred in the chest CTs of the test cohort while achieving significant reductions in scan length and DLP. Demircioğlu et al [18] successfully developed and validated a generative model for scan-range delineation of chest CT. Although their approach achieves robust results, it neglects the valuable diagnostic information available through segmentation masks, as studies have demonstrated that scout images can reveal clinically relevant findings not captured in the corresponding CT scan volumes [28]. For example, conditions such as cardiomegaly and aortic aneurysms could potentially be detected through analysis of segmentation masks derived from scout images [29].

As an additional proof-of-concept, we demonstrated that liver and skull segmentations allow for robust and precise planning of upper abdominal and cranial scans. This leads to a significant reduction in scan length and a low error rate. For the upper abdomen, even in challenging cases with ascites and right-sided pleural effusions, when clear delineation of the liver becomes more difficult.

Vendor-specific solutions for automated scan-range selection are available. Canon’s 3D Landmark Scan, for example, combines an ultra-low-dose helical CT with an AI-based anatomical landmark detection algorithm to determine the scan range, yielding a median total radiation dose reduction of 23.3% in chest and abdomen CT examinations [30]. To the best of our knowledge, it is the only commercially available software that is conceptually comparable to the algorithm proposed in this study. It is important to note, however, that it utilizes a helical scout acquisition unique to Canon systems. CT scanners from other vendors rely on standard 2D scout images, which are also used in our method. Tools aimed at improving the efficiency and accuracy of scan-range planning offered by GE (Smart Plan), Philips (Precise Planning), and Siemens (FAST Planning) instead rely on ceiling-mounted video cameras to detect external anatomical landmarks and suggest scan boundaries. These approaches remain only briefly described in publicly available sources, with no peer-reviewed validation and limited disclosure of their internal algorithms, training data, or performance characteristics. Our approach differs fundamentally. TOPOS produces explicit, multi-structure segmentations directly on standard 2D scout images across multiple scanner vendors, enabling transparent and verifiable scan-range definition based on the patient’s internal anatomy. As an illustrative example, a situs inversus case demonstrated that the model learned structure-specific features rather than being limited to simple spatial localization. This anatomy-driven approach allows radiographers to visually confirm the basis of automated planning, which can serve as a support system enhancing process transparency and workflow efficiency. supporting clinical acceptance and ensuring generalizability across multiple scan regions.

While our analysis accounted for various conditions suggesting robust applicability, in clinical practice, heterogeneous conditions are often present, which can compromise model accuracy. To partially mitigate this limitation, we evaluated the model on the publicly available CPTAC-LUAD dataset and found that segmentation performance for the chest target structures (aorta, heart, lung, trachea, thoracic spine) remained comparable. Moreover, in the subset of included CPTAC-LUAD chest examinations with available dose information, segmentation-derived scan-range planning yielded a significant reduction in scan length and DLP. This external validation supports robustness under dataset shift. Notably, 20 of the 36 external validation cases were acquired on GE Healthcare systems, while no scans acquired on systems from this vendor were included in model development. However, broader multicenter prospective testing across additional protocols and vendors remains necessary before clinical deployment. The study cohort was derived from a random 1% sample of all eligible examinations followed by manual quality control. This strategy preserved the real-world distribution of scanners, protocols, and patient characteristics, but required excluding cases with insufficient image quality. As a result, our cohort size was constrained by the manual review and annotation workflow, representing a trade-off between cohort size and annotation fidelity that may limit statistical power and generalizability. In addition, segmentation performance may be affected by anatomical superimposition inherent to 2D scout projections. For example, segmentation accuracy for the right kidney was lower than that of the left kidney, which is likely attributable to frequent overlap of the right kidney with the liver in anterior-posterior scout views. This superimposition can obscure organ boundaries and reduce contour visibility in the projection image. Such projection-related overlap is less pronounced on the left side, where adjacent structures typically provide clearer margins. While no single factor can be identified with certainty, anatomical overlap in the scout view represents a plausible explanation for this asymmetry. The effective dose as a measure of radiation exposure was not calculated, leaving uncertainty regarding the impact of automatic planning on biologically relevant radiation dose. While basic effective dose calculations for individual anatomical regions could be derived using conversion factors and the DLP [31], this would yield only approximate values, an approach we deliberately avoided given the availability of more sophisticated and accurate methods such as Monte Carlo simulations [32]. Segmentation-derived scan-range planning was evaluated only for anatomically standardized protocols (chest, upper abdomen, cranial), where required z-axis coverage can be defined unambiguously from organ extent. In indication-dependent protocols, scan extent is not determined by anatomy alone, and a single anatomical ground-truth for scan length is not well defined, limiting retrospective evaluation of planning accuracy and dose reduction. A major drawback of anatomy-based scan planning lies in complex scan regions such as CT neck, which may extend from the sinuses to the aortic arch depending on the examination. Planning based solely on the cervical spine would result in insufficient scan coverage. This limitation could potentially be addressed by a more granular set of anatomical target labels that better reflects indication specific scan boundaries.

In summary, our work demonstrates the applicability of an anatomical segmentation model trained on various target structures and scan regions for automatic scan planning for chest, upper abdominal and cranial CT, which significantly reduces radiation dose while maintaining a low error rate. Integrating such models into clinical practice has the potential not only to lower radiation exposure but also to reduce workload and optimize workflows, for example, by enabling real-time visualization of planning-relevant structures in the scout view.

## Supplementary information

Supplementary information

## Abbreviations

AI: Artificial intelligence
CT: Computed tomography
CTDI: Computed tomography dose index
DLP: Dose-length product
DSC: Dice-Sørensen coefficient
NIfTI: Neuroimaging Informatics Technology Initiative
NSD: Normalized surface distance
SD: Standard deviation
