# Supplementary material for: TOPOS: target organ prediction on scout views for automated CT scan planning
Source: Eur Radiol. 2026 May 14;36(10):7698–709. doi: 10.1007/s00330-026-12623-3 (PMC13574901; doi:10.1007/s00330-026-12623-3)
Supplement: Supplementary file 1 [file 330_2026_12623_MOESM1_ESM.pdf]

TOPOS: Target Organ Prediction of Scout Views for Automated CT Scan Planning

ELECTRONIC SUPPLEMENTARY MATERIAL

Supplementary Figure 1: Visualization of predicted Segmentation mask of the trained model in a scan with situs inversus.

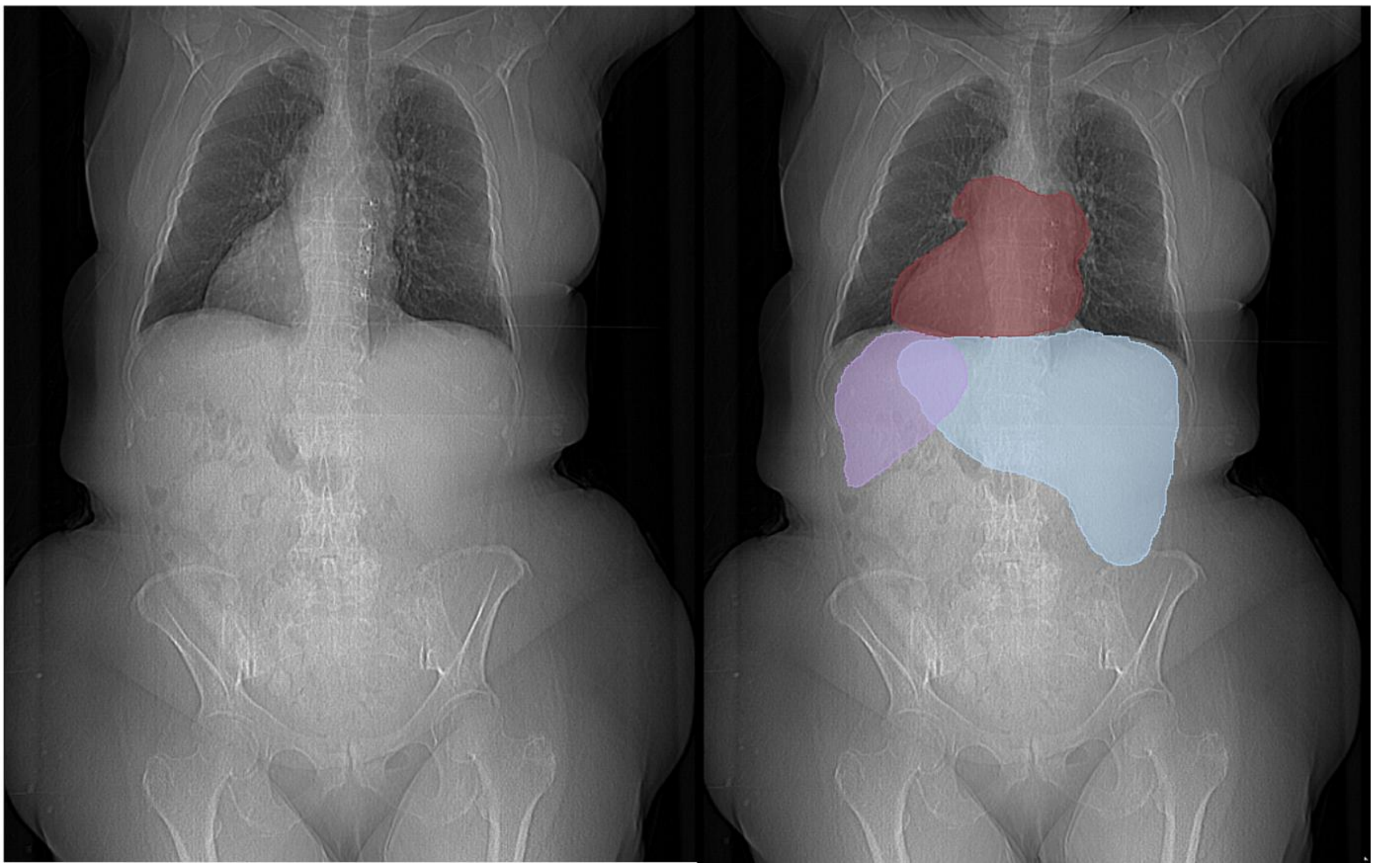

Supplementary Table 1: Segmentation labels of 26 anatomical structures and their corresponding substructures used for model development

Labels	**ID**	**Substructures**
Aorta	1	-
Clavicula (Left)	2	-
Clavicula (Right)	3	-
Colon	4	-
Femur (Left)	5	-
Femur (Right)	6	-
Heart	7	-
Hip (Left)	8	-
Hip (Right)	9	-
Humerus (Left)	10	-
Humerus (Right)	11	-
Kidney (Left)	12	-
Kidney (Right)	13	-
Liver	14	-
Lung	15	Lung Lower Lobe Left, Lung Lower Lobe Right, Lung Middle Lobe Right, Lung Upper Lobe Left, Lung Upper Lobe Right
Cervical Spine	16	Vertebrae C1, Vertebrae C2, Vertebrae C3, Vertebrae C4, Vertebrae C5, Vertebrae C6, Vertebrae C7
Thoracic Spine	17	Vertebrae T1, Vertebrae T2, Vertebrae T3, Vertebrae T4, Vertebrae T5, Vertebrae T6, Vertebrae T7, Vertebrae T8, Vertebrae T9, Vertebrae T10, Vertebrae T11, Vertebrae T12
Lumbar Spine	18	Vertebrae L1, Vertebrae L2, Vertebrae L3, Vertebrae L4, Vertebrae L5
Sacrum	19	-
Scapula (Left)	20	-
Scapula (Right)	21	-
Spleen	22	-
Stomach	23	-
Trachea	24	-
Brain	25	-
Skull	26	-

Supplementary Table 2: CT acquisition parameters across scanners and anatomical regions.

Scanner	Region	Tube voltage (kV)	Tube current (mAs)*	Collimation	Pitch	Reconstructed Slice-thickness
**Siemens Somatom Definition AS (64-row)**	Head	120	250–320	64 × 0.6 mm	0.8	3 mm
	Neck	100–120	120–180	64 × 0.6 mm	0.8	3 mm
	Chest	90–120	80–140	64 × 0.6 mm	0.8	3 mm
	Abdomen–Pelvis	100–120	120–200	64 × 0.6 mm	0.8	3 mm
	Chest–Pelvis	100–120	120–200	64 × 0.6 mm	0.8	3 mm
**Siemens Somatom Go.Top (128-row)**	Head	120	240–300	128 × 0.67 mm	0.8	3 mm
	Neck	100–120	100–160	128 × 0.67 mm	0.8	3 mm
	Chest	90–120	70–130	128 × 0.67 mm	0.8	3 mm
	Abdomen–Pelvis	100–120	120–180	128 × 0.67 mm	0.8	3 mm
	Chest–Pelvis	100–120	120–180	128 × 0.67 mm	0.8	3 mm
**Philips iCT (256-row)**	Head	120	280–340	256 × 0.625 mm	0.93	3 mm
	Neck	100–120	140–200	256 × 0.625 mm	0.93	3 mm
	Chest	100–120	80–140	256 × 0.625 mm	0.93	3 mm
	Abdomen–Pelvis	120	140–220	256 × 0.625 mm	0.93	3 mm
	Chest–Pelvis	120	140–220	256 × 0.625 mm	0.93	3 mm
**Philips IQon (256-row)**	Head	120	280–340	256 × 0.625 mm	0.98	3 mm
	Neck	100–120	140–200	256 × 0.625 mm	0.98	3 mm
	Chest	100–120	80–140	256 × 0.625 mm	0.98	3 mm
	Abdomen–Pelvis	120	140–220	256 × 0.625 mm	0.98	3 mm

	Chest–Pelvis	120	140–220	256 × 0.625 mm	0.98	3 mm
**Philips Incisive CT (128-row)**	Head	120	260–320	128 × 0.625 mm	0.93	3 mm
	Neck	100–120	120–180	128 × 0.625 mm	0.93	3 mm
	Chest	100–120	80–140	128 × 0.625 mm	0.93	3 mm
	Abdomen–Pelvis	120	120–200	128 × 0.625 mm	0.93	3 mm
	Chest–Pelvis	120	120–200	128 × 0.625 mm	0.93	3 mm
